# Genome‐Wide Association Studies Data and Transcriptomics Data Link Herpes Simplex Virus 1 Infection and Parkinson’s Disease

**DOI:** 10.1155/padi/4044371

**Published:** 2025-12-23

**Authors:** Changhao Lu, Xinyi Cai, Elena Simula, Tommaso Ercoli, Paolo Solla, Leonardo A. Sechi

**Affiliations:** ^1^ Department of Biomedical Sciences, University of Sassari, Sassari, Italy, uniss.it; ^2^ Department of Medical, Surgical and Experimental Sciences, University of Sassari, Sassari, Italy, uniss.it; ^3^ Department of Pathology, Shantou University Medical College, Shantou, China, stu.edu.cn; ^4^ Department of Neurology, University of Sassari, 07100, Sassari, Italy, uniss.it; ^5^ Struttura Complessa di Microbiologia e Virologia, Azienda Ospedaliera Universitaria di Sassari, 07100, Sassari, Italy, aousassari.it

**Keywords:** herpes simplex virus 1, herpesviral keratitis, nalfurafine, neuroinflammation, Parkinson’s disease

## Abstract

**Objective:**

The main goal of this study is to explore the link between herpes simplex virus 1 (HSV‐1) infection and Parkinson’s disease (PD) from the genome‐wide association studies (GWAS) data and find the shared molecular signature for mechanism understanding and drug repurposing from the transcriptomics data.

**Methods:**

We used summary‐level GWAS data for causal inference, exploring the association between herpes keratitis (mainly caused by HSV‐1) and PD, and used transcriptomics data to study the shared molecular signature for mechanism understanding and drug repurposing.

**Results:**

The causal inference analysis implied that HSV‐1 infection is related to PD. The upregulated shared gene set between HSV‐1 infection and PD is mainly enriched in neuroinflammation, while the downregulated shared gene set is mainly enriched in stem cell and cellular metabolism, and the drug repurposing targeted the shared molecular signature nalfurafine.

**Conclusion:**

HSV‐1 infection is related to PD, and these two diseases had shared molecular signature such as neuroinflammation and stem cell, which could be targeted for drug repurposing.

## 1. Introduction

Parkinson’s disease (PD) is defined by the degeneration of dopaminergic neurons in the substantia nigra [1], resulting in notable motor and nonmotor symptoms that gradually deteriorate over time [2, 3]. Despite numerous researches from the perspectives of mitochondrial dysfunction, impaired autophagy, oxidative stress, protein aggregation, neuroinflammation, environmental and genetic status [4], and treatment are currently only limited to symptom management, with no therapies available to heal the disease [5]. The need for new understanding using new methods is urgent.

Herpes simplex virus type 1 (HSV‐1) is a prevalent virus, infecting about 67% of the world population under the age of 50 [6]. The relationship between HSV‐1 and PD has been the focus of many studies with contradictory findings. Patients with idiopathic PD exhibited elevated levels of HSV‐1‐specific antibodies and a higher occurrence of HSV‐1 infections [7–11]. A population‐based case‐control study of incident PD among 2009 Medicare enrollees aged 66–90 (89,790 cases, 118,095 controls) revealed that the risk of PD was inversely associated with herpes simplex (OR 0.79, 95% CI 0.74–0.84) [12]. These contradictory results might partly be attributed to the heterogeneity of HSV‐1 infections and the limitations in observational studies, such as confounding factors [13], selection bias [14], and reverse causation [15].

Herpes keratitis is mainly caused by HSV‐1 direct infection or its reactivation from latency to cornea and conjunctiva [16]. Although the study of the relationship between HSV‐1 and PD has been the focus of many studies, the relationship between herpes keratitis as a specific HSV‐1 infection and PD has not been explored. Investigating the relationship between herpes keratitis as a specific HSV‐1 infection and PD, rather than broadly studying the relationship between HSV‐1 and PD, has scientific advantages. Focusing on one specific HSV‐1 infection could reduce confounding factors from other HSV‐1 infections, therefore providing more accurate conclusion.

As for the limitations in observational studies, randomized controlled trials (RCTs) could address these issues because the random assignment of participants to exposure helps to ensure that all the confounding factors are evenly distributed across all groups. However, in the case of human herpes keratitis and PD, RCTs are not ethical nor feasible. Mendelian randomization (MR), as an alternative method to RCTs, uses single nucleotide polymorphism (SNP) as instrumental variables (IVs), which are randomly assigned at conception, independent of the confounding factors. By using MR, the causal relationship between herpes keratitis and PD could be tested without ethical concerns and the limitation inherent in observational studies. Aside from using MR as a strong tool to infer causal relationship between herpes keratitis and PD, we used transcriptomics data to study the potential shared molecular signature between herpes keratitis and PD, which is important to mechanism understanding and drug repurposing.

Our study proposed that HSV‐1, specifically in the form of herpes keratitis, could be a causal risk factor to PD through specific molecular mechanisms. We chose a specific HSV‐1 infection, herpes keratitis, to avoid the confounding effect of the heterogeneity in HSV‐1 infections, and we chose MR for its unbiased estimates of causal effects, which made it deal for clarifying the controversial relation, and transcriptomics analysis allowed us to capture the shared molecular feature that might help us understand the relation between these two diseases in a new perspective and lay the foundation for drug repurposing. We aimed to answer the following questions: is there a causal relationship between herpes keratitis and PD? what are the shared molecular signatures? how might these findings aided in the drug repurposing? by addressing these critical questions, our study not only clarified the association between herpes keratitis and PD but also found the shared molecular signature for mechanism understanding and drug repurposing, offering hope for better therapeutic outcomes.

## 2. Materials and Methods

### 2.1. Study Design

**Figure 1:**
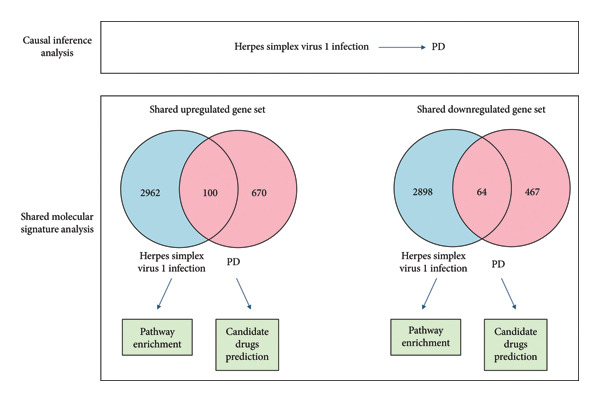
The study flowchart presents an integration of GWAS data and transcriptomics to study the relationship between HSV‐1 and PD.

All data used in this study were obtained from the publicly accessible GWAS database without the need for re‐ethical approval. We performed a causal inference using bidirectional MR to test the causality between herpes keratitis and PD, and the pathway analysis and drug repurposing using shared molecular signatures were performed. The study workflow is depicted in Figure 1.

### 2.2. GWAS Data Source

The GWAS data for this research were sourced from publicly accessible databases (Supporting Table S1 for details). Herpes keratitis GWAS data were extracted from FinnGen R10 study [17], the work led by Kurki et al. encompassing 1252 cases and 390,647 controls. PD data were obtained from the work led by Nalls et al. [18], including 33,674 cases and 449,056 controls. Notably, the GWAS datasets were acquired from two sample, ensuring no overlap among data.

### 2.3. Source of PD Transcriptomics Differential Expression Data

The PD case versus control differential expression data in PPMI came from the work of Craig and collaborators, where genetics background, age groups, and sex were considered [19–21]. This set of bulk RNAseq data came from 4871 longitudinally collected whole‐blood samples of 1570 PD cases. The assessment of differential expression with adjusted *p* value of less than 0.05 and a log2 fold change greater than 0.1 was detailed in the paper of Craig and collaborators.

### 2.4. HSV‐1‐Infected Fibroblast KMB17 as a Model for Transcriptomics Differential Expression Data

We used HSV‐1‐infected fibroblast KMB17 transcriptomics data as a model for HSV‐1 infection, marked by accession number GSE103763. Differential expression analysis was conducted using the GEO2R [22] online tool (https://www.ncbi.nlm.nih.gov/geo/geo2r/). We employed log2 fold change greater than 1 and adjusted *P* values of less than 0.05 to filter for genes with significant expression differences as seen in Supporting Table S8.

### 2.5. Instrumental Variables Selection and Data Harmonization

According to the principles of MR [23], IVs [24] should be SNPs that closely related to the exposure. We screened SNPs with significant correlation with herpes keratitis and PD. Subsequently, we conducted linkage disequilibrium (LD) analysis on the SNPs, using a 10,000 kb window and a r2 value cutoff of less than 0.001, to exclude the mutual linkage SNPs and nonbiallelic SNPs. SNPs exhibiting an F‐statistic over 10 were selected to mitigate mild instrument bias in MR analyses [25]. Furthermore, to ensure the selected SNPs are consistent on effect direction and alleles across two samples, “harmonise_data” function is used to check for SNPs with inconsistent directions, palindromic SNPs, and SNPs with mismatched frequencies [26].

### 2.6. Bidirectional Two‐Sample MR

For bidirectional two‐sample MR, herpes keratitis and PD were studied alternately as exposure and outcome. We applied several MR methods to each direction of estimation. All analyses were performed based on the “TwoSampleMR” R package [27, 28]. The inverse variance weighted method (IVW) is the main MR approach which utilized meta‐analytical methods to consolidate the Wald ratios indicative of the causal effects linked to each individual SNP [29, 30]. The weighted median method is a robust causal estimate method. Weights for each SNP were calculated based on the inverse of the standard error of its effect size. The simple mode method selects the most common causal estimate mode observed across a set of IVs as the final causal effect estimate. While the weighted mode method integrates the weights calculated for each SNP based on the inverse of the standard error of its effect size and uses the weighted median estimator to provide a robust causal estimate. Bayesian weighted median regression (BWMR) uses Bayesian approaches combined with weighted median estimates to provide a more flexible and informative statistical estimate of the causal effect [31]. The strength of the causal inference was evaluated by Cochran’s Q statistics, MR‐Egger intercept tests, and leave‐one‐out sensitivity analyses [32]. Cochran’s Q statistics evaluated heterogeneity in IVW models [33]. The MR‐Egger regression is used to detect and adjust for pleiotropy bias of genetic instruments. It introduces an intercept to assess the average pleiotropic effects across IVs and allows for the IVs’ effects to be partially independent of the exposure [34]. A nonzero intercept in the MR effect estimates of IVs implies the existence of horizontal pleiotropy [35].

### 2.7. Pathway Enrichment Analysis

Pathway enrichment analysis was performed for the shared upregulated gene set and shared downregulated gene set among herpes keratitis and PD, using Metascape (http://metascape.org) [36]. The analysis included KEGG pathway, GO biological processes, reactome gene sets, and others. The minimum overlap, *p*‐value cutoff, and enrichment factor settings were kept at default values provided by Metascape, ensuring a strict filtering of statistically significant pathways, and the bar plot was drawn by “ggplot2” R package.

### 2.8. Drug‐Repurposing Analysis Using IDG Drug Target 2022

To explore the potential drug targets associated with the shared upregulated gene set and shared downregulated gene set among herpes keratitis and PD, the Enrichr [37] (https://maayanlab.cloud/Enrichr/) was used. Specifically, we used the Illuminating the Druggable Genome (IDG) Drug Target 2022 library, which incorporated the latest information on drug targets collected from the up‐to‐date genomic and drug research [38]. The results were determined by the adjusted *p* value (< 0.05) provided by Enrichr.

## 3. Results

### 3.1. Testing the Unidirectional Causal Effect of Herpes Keratitis on PD

We extracted 45 SNPs as IVs from the herpes keratitis GWAS dataset, details including *p* values, beta coefficients, and standard errors and the effect allele can be seen in Supporting Table S2, and we selected 112 SNPs as IVs from the PD GWAS dataset, details seen in Supporting Table S3. Furthermore, we calculated the F‐statistic for each SNP, which all exceeded 10. Using IVW as the primary method, our analysis revealed a causal influence of herpes keratitis on PD. This suggested that herpes keratitis is associated with a higher odd of developing PD, details seen in Figure 2(a). This result was validated by BWMR, and complemented by MR Egger, weighted median, simple mode, and weighted mode, details seen in Supporting Figure S2. Characteristic of the herpes keratitis‐related genetic variants and their effects on PD is detailed in Supporting Table S4. Characteristic of the PD‐related genetic variants and their effects on herpes keratitis is detailed in Supporting Table S5. Effect estimates of the associations of herpes keratitis and Parkinson’s disease after excluding outliers SNPs are detailed in Supporting Table S6. The MR‐Egger regression analysis showed an Egger intercept of 0.006230545 with a nonsignificant *p* value of 0.55215, suggesting no evidence of horizontal pleiotropy. The MR‐PRESSO global test showed a residual sum of squares (RSSobs) of 56.03422 with a nonsignificant *p* value of 0.163, suggesting no horizontal pleiotropy. The IVW heterogeneity test showed a Q‐value of 52.90191 with a nonsignificant *p* value of 0.16812, suggesting no heterogeneity among the IVs. The MR‐Egger heterogeneity test showed a Q‐value of 52.46377 with a nonsignificant *p* value of 0.15275, also suggesting no heterogeneity. The symmetric funnel plots supported the lack of substantial bias in SNP selection, as seen in Supporting Figure S1(A). The scatter plot illustrated the causal association between herpes keratitis and PD, shown by the slope of the line, which varies based on the MR tests, as seen in Supporting Figure S2(B). The causal relationship between herpes keratitis and PD is evaluated using IVW methods for each specific SNP, as seen in Supporting Figure S1(C). The forest plot illustrated the leave‐one‐out analysis. Each dot represented the MR estimate result using IVW that excludes the particular SNP, as seen in Supporting Figure S1(D). We further explored the causal effect of PD on herpes keratitis. Using IVW as the primary method, our analysis revealed no causal influence of PD on herpes keratitis, details shown in Figure 2(b). This outcome was verified by BWMR and supplemented by MR Egger, weighted median, simple mode, and weighted mode, details shown in Supporting Figure S3. The assessment of heterogeneity and horizontal pleiotropy using various methods could be found in Supporting Table S7. The MR‐Egger regression analysis showed an Egger intercept of −0.001123141 with a nonsignificant *p* value of 0.912001, suggesting no evidence of horizontal pleiotropy. The MR‐PRESSO global test showed RSSobs of 102.3366 with a nonsignificant *p* value of 0.735, suggesting no horizontal pleiotropy. The IVW heterogeneity test showed a Q‐value of 99.99259 with a nonsignificant *p* value of 0.764103, suggesting no heterogeneity among the IVs. The MR‐Egger heterogeneity test showed a Q‐value of 99.98032 with a nonsignificant *p* value of 0.742762, also suggesting no heterogeneity.

Figure 2Forest plot of bidirectional MR results using IVW and BWMR. (a) Using IVW and BWMR to test the causal relationship between herpes keratitis (exposure) and PD (outcome). IVW: OR = 1.0484, 95% CI = 1.0119–1.0863, p = 0.0090∗; BWMR: OR = 1.0565, 95% CI = 1.0140–1.1008, p = 0.0087∗. (b) Using IVW and BWMR to test the causal relationship between PD (exposure) and herpes keratitis (outcome). IVW: OR = 1.0098, 95% CI = 0.9427–1.0817, p = 0.7808; BWMR: OR = 1.0185, 95% CI = 0.9451–1.0976, p = 0.6307.∗: p < 0.05.

**Figure:**
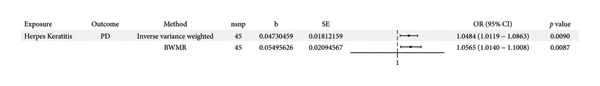
(a)

**Figure:**
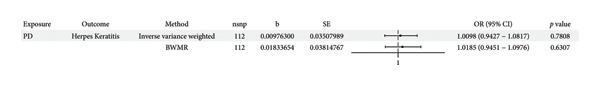
(b)

### 3.2. Exploring Shared Molecular Signatures Between Herpes Keratitis and PD

The PD case versus control differential expression data in PPMI came from the work of Craig and collaborators, and we used HSV‐1 infected fibroblast KMB17 transcriptomics data as a model for herpes keratitis. Overall, 100 shared upregulated genes between herpes keratitis and PD were found as in Figure 3(a), detail seen in Supporting Table S9, and the pathway analysis on the shared upregulated gene set was mainly enriched in “G0:0043122: regulation of canonical NF‐kappaB signal transduction,” “M166: PID ATF2 PATHWAY,” “G0:0002718: regulation of cytokine production involved in immune response,” “R‐HSA‐6798695: neutrophil degranulation,” and “R‐HSA‐375165: NCAM signaling for neurite out‐growth,” as in Figure 3(c), detail seen in Supporting Table S10. 64 shared downregulated genes between herpes keratitis and PD are found as in Figure 3(b), detail seen in Supporting Table S9, and the pathway analysis on the shared downregulated gene set was mainly enriched in “WP3888: VEGFA VEGFR2 signaling,” “GO:0072089: stem‐cell proliferation,” “G0:0034764: positive regulation of transmembrane transport,” “G0:0010720: positive regulation of cell development,” and “G0:0044089: positive regulation of cellular component biogenesis,” as in Figure 3(d), detail seen in Supporting Table S11.

Figure 3Shared molecular signatures analysis between herpes keratitis and PD. (a) Shared upregulated gene set. (b) Shared downregulated gene set. (c) Pathway analysis on the shared upregulated gene set. (d) Pathway analysis on the shared downregulated gene set.

**Figure:**
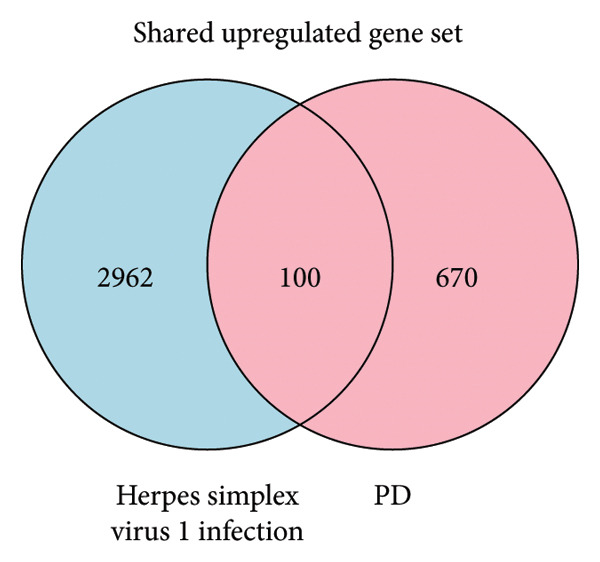
(a)

**Figure:**
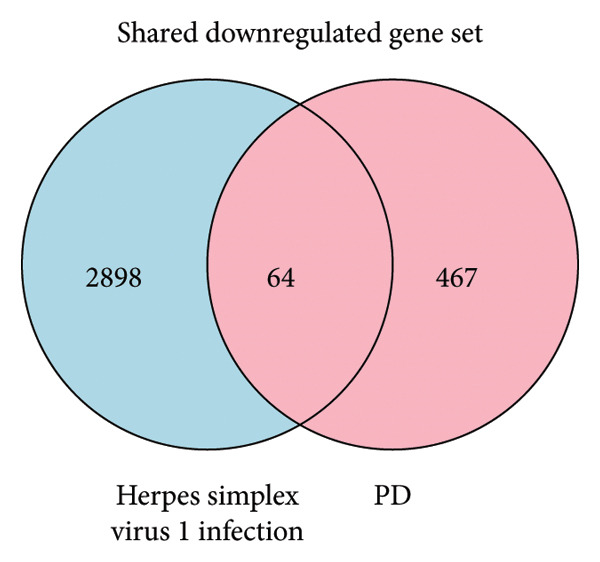
(b)

**Figure:**
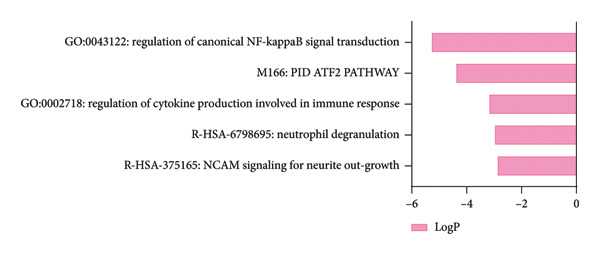
(c)

**Figure:**
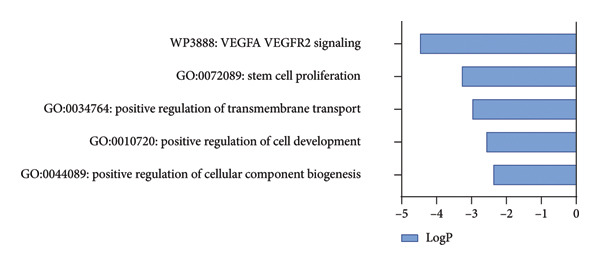
(d)

### 3.3. Drug‐Repurposing Analysis Using IDG Drug Target 2022

We explored the potential drug targets associated with the shared upregulated gene set and shared downregulated gene set among herpes keratitis and PD, using the Enrichr. Specifically, we used the IDG Drug Target 2022 library, which incorporated the latest information on drug targets collected from the up‐to‐date genomic and drug research. The results were determined by the adjusted *p* value (< 0.05) provided by Enrichr. The top 10 drugs targeting shared upregulated gene set between herpes keratitis and PD, ranked by minimum adjusted *P* value, as shown in Table 1, detail seen in Supporting Table S12, and the top 10 drugs targeting shared downregulated gene set between herpes keratitis and PD, ranked by minimum adjusted *P* value, as shown in Table 2, detail seen in Supporting Table S13. The results showed that nalfurafine is the only drug molecule that interacted with the shared upregulated gene set among herpes keratitis and PD.

**Table 1:** List of top 10 drugs targeting shared upregulated gene set between herpes keratitis and PD, ranked by minimum adjusted *P* value.

Term	Gene	*p* value	Adjusted *P* value
Nalfurafine	ACHE; CACNA1F	0.0062	0.0368
Cisplatin	ACHE; NT5E; SLC2A4	0.0066	0.0681
Tannic acid	CACNA1F	0.0159	0.0681
Zafirlukast	SLC12A3	0.0159	0.07031
Nelfinavir	ACHE	0.0159	0.07031
Sulconazole	ACHE	0.0159	0.1032
Hexachlorophene	SLC12A3	0.0159	0.1077
Bosutinib	SLC12A3	0.0159	0.1273
Sorafenib	SLC12A3	0.0159	0.1273

**Table 2:** List of top 10 drugs targeting shared downregulated gene set between herpes keratitis and PD, ranked by minimum adjusted *P* value.

Term	Gene	*p* value	Adjusted *p* value
Nifedipine	ACHE; CACNA1F	0.0062	0.0828
Quercetin	ACHE; NT5E; SLC2A4	0.0066	0.0828
(S)‐Nitrendipine	CACNA1F	0.0159	0.0828
Cyclothiazide	SLC12A3	0.0159	0.0828
Alfuzosin	ACHE	0.0159	0.0828
Stearic acid	ACHE	0.0159	0.0828
Hydrochlorothiazide	SLC12A3	0.0159	0.0828
Bendroflumethiazide	SLC12A3	0.0159	0.0828
Benzthiazide	SLC12A3	0.0159	0.0828

## 4. Discussion

Although extensive research effort already put on the theory of mitochondrial dysfunction, impaired autophagy, oxidative stress, protein aggregation, neuroinflammation, and environmental and genetic status, current treatments for PD are still limited to symptom management and are unable to heal the disease. Therefore, it is urgent to expand our understanding using new methods from new another perspective. Von Economo was the first researcher to propose a possible correlation between viral infections and PD; he found that the lethargic encephalitis was associated with the inflammation in the midbrain tegmentum and substantia nigra, which is linked to PD [39]. Subsequent research demonstrated that a highly pathogenic H5N1 influenza virus may infiltrate the central nervous system, resulting in neuroinflammation and neurodegeneration, including PD [40]. Among the studies on viral infections and PD, many have pointed toward a complex relation between HSV‐1 infection and PD. Marttila R.J. and collaborators found an increased level of IgG antibodies against HSV‐1 in the PD group [7]. Marttila R. and collaborators found that PD patients had higher levels of serum antibodies against HSV‐1 compared to controls, suggesting a possible immune response to the virus. However, cerebrospinal fluid (CSF) analyses show no changes of antibodies against HSV‐1 compared to controls, suggesting that HSV‐1 might influence PD through inflammation rather than direct nervous system infection [9]. Agostini and collaborators found that higher serum HSV‐1‐specific antibody titers were seen in PD patients compared to HC, and the polymorphisms of the HSV‐1 infection‐related gene PILRA was involved, suggesting HSV‐1 might influence PD through neuroinflammation [41]. However, Camacho‐Soto A and collaborators found that rather than contributing to the development of PD, HSV‐1 and ‐2 infections might actually be associated with a reduced risk of PD, based on a population‐based case‐control study involving 2009 Medicare beneficiaries aged 66–90 years (89,790 cases, 118,095 randomly selected comparable controls), suggesting future studies to test if this inverse association is causal [12]. These contradictory results on the complex relation between HSV‐1 infection and PD might partly be attributed to the heterogeneity of HSV‐1 infections and the limitations in observational studies, such as confounding factors [13], selection bias [14], and reverse causation [15]. The global seroprevalence of HSV‐1 is above 60%, and almost all adults over 50 have been exposed to the varicella zoster virus (VZV), although the lifetime prevalence of PD is still low [42, 43]. This difference means that only a limited group of people who are vulnerable may move from being exposed to a virus to having their nerves break down. A recent meta‐analysis indicated that herpes zoster is associated with a marginally elevated risk of Parkinson’s disease (HR = 1.15, 95% CI 1.03–1.30), reinforcing the hypothesis that viral reactivation may serve a contribution rather than a deterministic function [44].

To reduce the heterogeneity effect of HSV‐1 infections, our study focused on one specific HSV‐1 infection, herpes keratitis, and to test the causal relationship between herpes keratitis and PD, we used bidirectional MR for causal inference. 45 SNPs were extracted from the herpes keratitis GWAS dataset as IVs. The IVs we chose met the three main assumptions (relevance, independence, and exclusion restriction) in MR. First, to meet the relevance assumption, the selected IVs were strongly correlated with the exposure, tested using F‐statistic greater than 10, reducing the weak instrument biases. Second, to meet the independence assumption, multiple IVs were selected from GWAS data, which were not associated with confounding factors. Third, to meet the exclusion restriction assumption, MR‐Egger regression and leave‐one‐out analysis were performed. MR‐Egger regression tested the direct effect of IVs on the outcome by the intercept. If the intercept is significantly different from zero, it shows the evidence of horizontal pleiotropy, violating the exclusion restriction. The leave‐one‐out analysis involves recalculating the estimate multiple times, each time omitting one of the IVs. If the estimates vary widely with the exclusion of one specific IVs, it indicates the violation of the exclusion restriction. The IVs themselves are not directly related to the outcomes. In MR, IVs are used only to test the causal relationships between exposures and outcomes.

With 45 IVs selected from herpes keratitis GWAS dataset, the IVW as the main method showed a causal influence of herpes keratitis on PD, suggesting that herpes keratitis is associated with a higher odd (about 4.8%) of developing PD. The robustness of our findings is supported by complementary MR methods, especially the BWMR method, which also indicated the causal effect of herpes keratitis on PD. The MR‐Egger regression analysis and MR‐PRESSO global test showed no evidence of horizontal pleiotropy. The IVW and MR‐Egger heterogeneity test showed no heterogeneity among the IVs. In a similar process, with 112 IVs selected from the PD GWAS dataset, our analysis revealed no causal influence of PD on herpes keratitis. Our study indicated the unidirectional causal effect of herpes keratitis on PD. Our study showed the first evidence of a unidirectional causal relationship between herpes keratitis, primarily induced by HSV‐1 infection, and PD. This causal inference discovery added new evidence to the viral infection theory of the PD’s etiology.

Our transcriptomic analyses provided further insight into the shared molecular features between herpes keratitis and PD. The PD case versus control differential expression data in PPMI was derived from the research by Craig  and collaborators. Additionally, we used transcriptomics data from HSV‐1‐infected fibroblast KMB17 as a model for herpes keratitis. We found 100 shared upregulated genes and 64 shared downregulated genes, which showed molecular bases for the shared molecular features between herpes keratitis and PD.

The patients’ genetic backgrounds substantially influence the clinical outcomes and the age of onset of PD [20, 21]. Certain variants influence clinical trajectories differently: alleles in the GBA gene (e.g., p.E326K, p.N370S, and p.T369M) correlate with varying rates of motor and cognitive decline, whereas variants in MAPT and SNCA are associated with disparities in motor progression and disease severity. LRRK2 mutations yield diverse outcomes; specifically, G2019S carriers frequently exhibit a more gradual decline in motor function relative to R1441C carriers, which operate via distinct molecular pathways. Cumulative genetic risk scores (GRS), calculated from the additive effects of various SNPs, have demonstrated the ability to forecast the pace of disease progression. Individuals with elevated GRS values attain Hoehn and Yahr stage 3 more swiftly, demonstrating an earlier age of onset in comparison to those with lower scores [20]. Overall, these results show that uncontrolled genetic diversity in trial groups can skew results. The datasets utilized in our study already address the concern regarding genetic background and age of onset. The PD case versus control differential expression data in PPMI came from the work of Craig  and collaborators, where genetics background and age groups were considered [19]. So we are confident that our observed associations between HSV‐1 infection and PD are not confounded by genetic background.

Sex related differences in PD are recognized across epidemiology, clinical presentation, and molecular level [45]. Epidemiologically, men exhibit roughly 1.5–2 times higher prevalence and incidence than women, with onset occurring on about 2.2 years earlier and mortality risk being slightly higher in males [46–49]. Clinical presentations also differ: women more often present with tremor‐dominant disease and milder early motor impairment, whereas men develop greater rigidity, gait abnormalities, and postural issues [50, 51]. Nonmotor features also diverge. Women with PD report more anxiety, depression, fatigue, pain, and dysautonomia, while men more frequently show orthostatic hypotension, urinary dysfunction, and REM sleep behavior disorder [52–54]. Cognitive outcomes follow a similar sex bias: male PD patients are at higher risk of cognitive decline and dementia, often with deficits in executive function and verbal fluency, whereas women are relatively protected, though they may present more visuospatial deficits [55]. At the molecular level, estrogen enhances dopaminergic resilience through mitochondrial stabilization, anti‐inflammatory signaling, and modulation of *α*‐synuclein aggregation, while its decline after menopause coincides with rising female PD incidence [48, 56–60]. While sex differences are critical in PD, the PD case versus control differential expression data in PPMI came from the work of Craig  and collaborators, where sex factor was considered [19]. So we are confident that our observed associations between HSV‐1 infection and PD are not confounded by sex background.

Our study of pathway analysis on the shared upregulated gene set was mainly enriched in “G0:0043122: regulation of canonical NF‐kappaB signal transduction,” “M166: PID ATF2 PATHWAY,” and “G0:0002718: regulation of cytokine production involved in immune response.” These results provided critical insights into the potential mechanisms by which herpes keratitis might influence the PD, highlighting the inflammatory processes. The NF‐κB signaling pathway plays an important role in both herpes keratitis and PD. In the HSV‐infected human corneal epithelial cells, activated NF‐κB signaling is associated with proinflammatory cytokines such as IL‐6, IL‐8, and TNF‐α [61]. In PD, NF‐κB signaling is associated with NLRP3 inflammasome and Notch signaling in microglia, which releases the proinflammatory cytokines and exacerbates neuroinflammation, promoting neuronal damage [62–64]. The p38MAPK/ATF‐2 pathway is associated with the survival of neuronal cells and inflammation [65]. Cytokine production in the immune response is important to both herpes keratitis and PD. In herpes keratitis, IL‐17 binding to TNF‐α or IFN‐γ may stimulate the synthesis of IL‐6, IL‐8, and MIP3‐α, leading to the progression of inflammation [66]. The presence of chemokines in the cornea infected with HSV‐1 significantly affects the inflammatory cell infiltration into the cornea [67]. In PD, levels of serum cytokines, including IL‐1ß, IL‐2, IL‐10, IFNγ, and TNF‐α, have been shown to be associated with the severity and progression of PD symptoms [68–72]. Vitiligo is similar to PD, as another type of melanin‐containing cell (melanocytes) is damaged in vitiligo; both melanocytes and dopaminergic neurons are melanin‐containing cells and may share vulnerabilities to immune‐mediated damage because of HSV‐1 infection [73–75].

Our study of pathway analysis on the shared downregulated gene set was mainly enriched in “WP3888: VEGFA VEGFR2 signaling,” “GO:0072089: stem‐cell proliferation,” and “G0:0034764: positive regulation of transmembrane transport.” These results provided critical insights into the potential mechanisms by which herpes keratitis might influence the PD, highlighting several pathways that are crucial for cellular maintenance and development. Astrocytic‐derived VEGFA is crucial in PD for its involvement in the breakdown of the blood–brain barrier (BBB), which is essential for normal brain function, synaptic remodeling, and angiogenesis [76]. The enrichment of stem‐cell proliferation implies the essence of PD. PD is defined by the progressive loss of dopaminergic neurons in the substantia nigra, with the accumulation of Lewy bodies and a decline in the neural regenerative capacity [77, 78].

Our analysis identified nalfurafine as the only potential therapeutic drug molecule that interacts with the shared upregulated genes between herpes keratitis and PD. As a kappa‐opioid receptor agonist [79], nalfurafine was synthesized by structural modification of the opioid antagonist naltrexone. It is an antipruritic medication used in Japan to treat uremic pruritus in persons with chronic kidney disease who are receiving hemodialysis [80]. It was the first selective KOR agonist that was authorized for clinical usage [81, 82]. Recent study found that nalfurafine could alleviate herpetic and post‐herpetic pain dose‐dependently in mice [80], and the application of nalfurafine directly onto the cornea effectively reduces neovascularization and inflammation [83]. Nalfurafine has shown effect on neuroprotection [84], remyelination [79], and reduction of levodopa‐induced dyskinesia [85]. Combined with our study, it is probable that nalfurafine could be used for pain and inflammation management in herpes keratitis patients and for the management of the PD risk.

Our findings about therapeutic implications are hypothesis generating. No randomized controlled studies (RCTs) that look into whether antiviral therapy or the herpes vaccine have an influence on PD. Nonetheless, observational data collected by Lehrer and Rheinstein (2022) revealed that U.S. states with a greater frequency of Parkinson’s disease had lower shingles vaccination rates among those aged ≥ 60, and vaccination was associated with a reduced risk of dementia [86]. These observational correlations are unable to establish causality, and the mentions of antivirals or *κ*‐opioid receptor agonists (such as nalfurafine) in this paper are possible future directions, not clear clinical guidance.

## 5. Conclusions

This study establishes a causal link between herpes keratitis and PD using GWAS and transcriptomic data, and these two diseases had shared molecular signature such as neuroinflammation and stem cell. Additionally, our findings highlighted nalfurafine as a promising drug‐repurposing candidate.

## Ethics Statement

The authors have nothing to report.

## Consent

The authors have nothing to report.

## Disclosure

All authors have read and agreed to the published version of the manuscript.

## Conflicts of Interest

The authors declare no conflicts of interest.

## Author Contributions

Conceptualization: Leonardo A Sechi; methodology, software, validation, formal analysis, investigation, and data curation: Changhao Lu, Xinyi Cai, Tommaso Ercoli, and Elena Rita Simula; writing–original draft preparation: Changhao Lu and Xinyi Cai; writing–review and editing: Tommaso Ercoli, Leonardo A. Sechi, and Paolo Solla; supervision: Leonardo A. Sechi and Paolo Solla; project administration: Changhao Lu, Xinyi Cai, Tommaso Ercoli, Leonardo A. Sechi, and Paolo Solla.

## Funding

This study was funded by Regione Autonoma Sardegna grant legge regionale 12 22 December 2022 n. 22 and Ministero Università e Ricerca PRIN 2022 n: 2022BP837R. Open access publishing facilitated by Universita degli Studi di Sassari, as part of the Wiley ‐ CRUI‐CARE agreement.

## Supporting Information

Additional supporting information can be found online in the Supporting Information section.

## Supporting information


**Supporting Information 1** Supporting Figure 1: relationship examination between herpes keratitis and PD. (A) Funnel plot was used to illustrate the individual variation effects for the instrument variables shown against the inverse of their standard error. (B) Scatter plot depicted the causal relationship between herpes keratitis and PD by the line’s slope, which varies depending on the MR tests. (C) Forest plot was used to illustrate how herpes keratitis is associated with an increased risk of PD. (D) The causal association between herpes keratitis and PD is assessed by IVW approaches for each individual SNP.

**Supporting Information 2** Supporting Figure 2: forest plot of MR results to test the causal relationship between herpes keratitis (exposure) and PD (outcome), using MR Egger, weighted median, simple mode, and weighted mode.

**Supporting Information 3** Supporting Figure 3: forest plot of MR results to test the causal relationship between PD (outcome) and herpes keratitis (exposure), using MR Egger, weighted median, simple mode, and weighted mode.

**Supporting Information 4** Supporting Table S1: detailed information for database.

**Supporting Information 5** Supporting Table S2: instrumental variables used in MR analysis in herpes keratitis.

**Supporting Information 6** Supporting Table S3: instrumental variables used in MR analysis in Parkinson’s disease.

**Supporting Information 7** Supporting Table S4: characteristic of the herpes keratitis‐related genetic variants and their effects on PD.

**Supporting Information 8** Supporting Table S5: characteristic of the PD‐related genetic variants and their effects on herpes keratitis.

**Supporting Information 9** Supporting Table S6: effect estimates of the associations of herpes keratitis and Parkinson’s disease after excluding outliers SNPs.

**Supporting Information 10** Supporting Table S7: evaluation of heterogeneity and horizontal pleiotropy using different method.

**Supporting Information 11** Supporting Table S8: GSE103763‐mRNA.

**Supporting Information 12** Supporting Table S9: shared upregulated gene set and shared downregulated gene set.

**Supporting Information 13** Supporting Table S10: pathway analysis on the shared upregulated gene set.

**Supporting Information 14** Supporting Table S11: pathway analysis on the shared downregulated gene set.

**Supporting Information 15** Supporting Table S12: drugs targeting shared upregulated gene set between herpes keratitis and PD, ranked by minimum adjusted *p* value.

**Supporting Information 16** Supporting Table S13: drugs targeting shared downregulated gene set between herpes keratitis and PD, ranked by minimum adjusted *p* value.

## Data Availability

All data used in this study are publicly available from the following databases: PPMI database (https://www.ppmi-info.org/data), GWAS catalog (https://www.ebi.ac.uk/gwas/), and GSE103763. For up‐to‐date information on the study, visit https://www.ppmi-info.org/.
