# Supplementary figures and images for: Genome‐Wide Association Studies Data and Transcriptomics Data Link Herpes Simplex Virus 1 Infection and Parkinson’s Disease
Source: Parkinsons Dis. 2025 Dec 23;2025:4044371. doi: 10.1155/padi/4044371 (PMC12767429; doi:10.1155/padi/4044371)

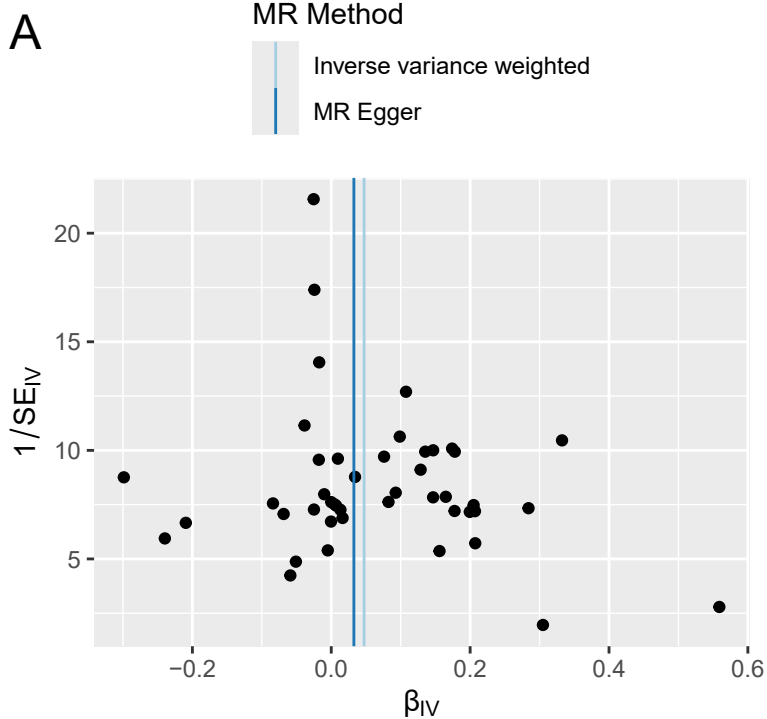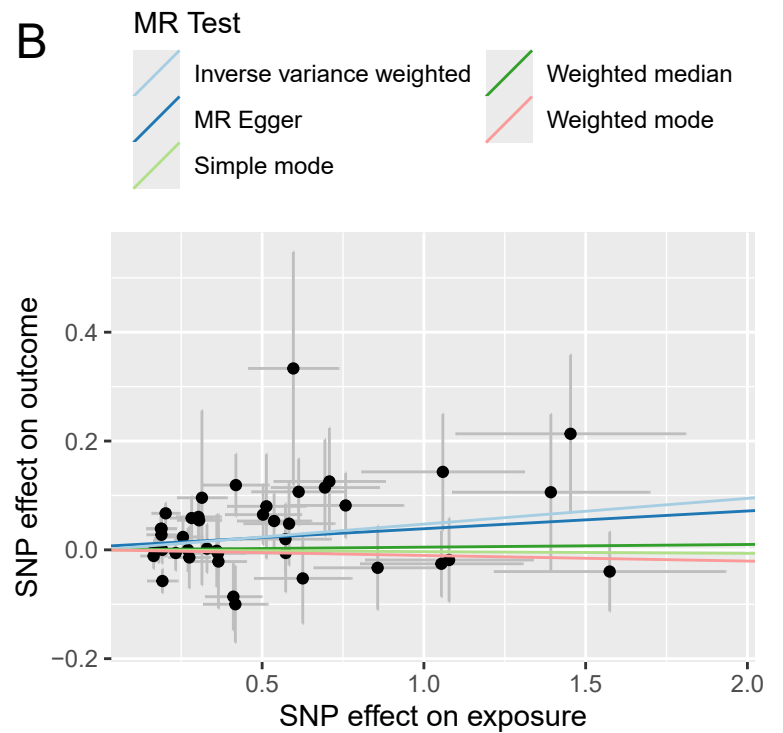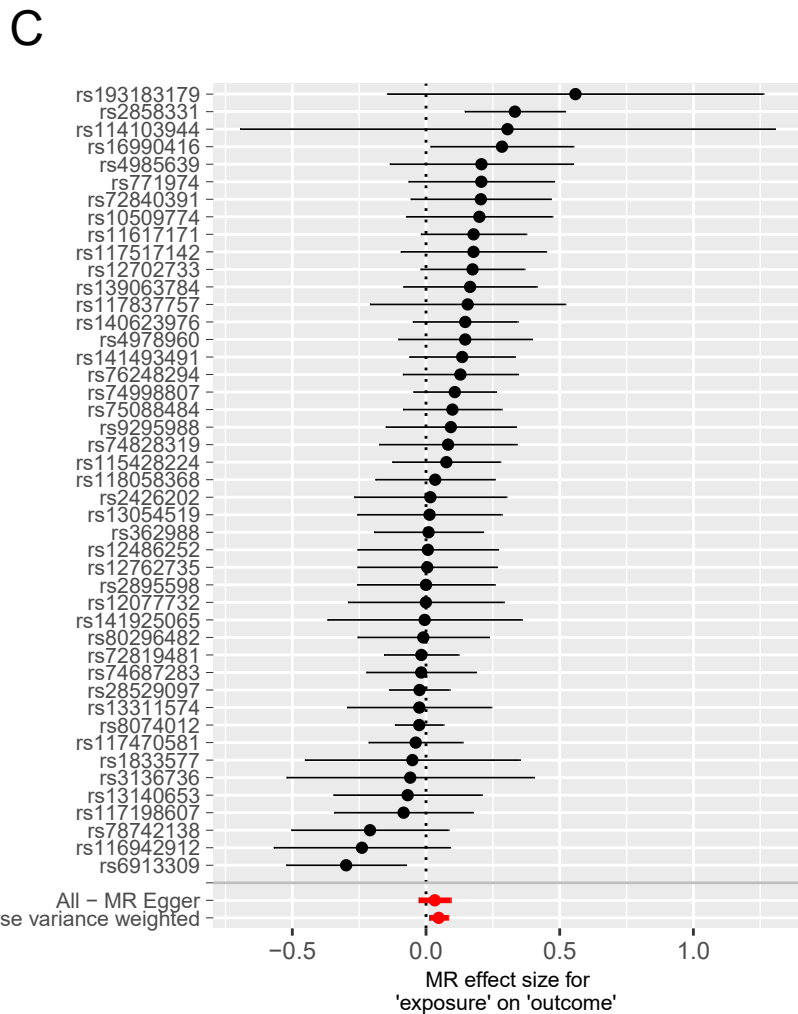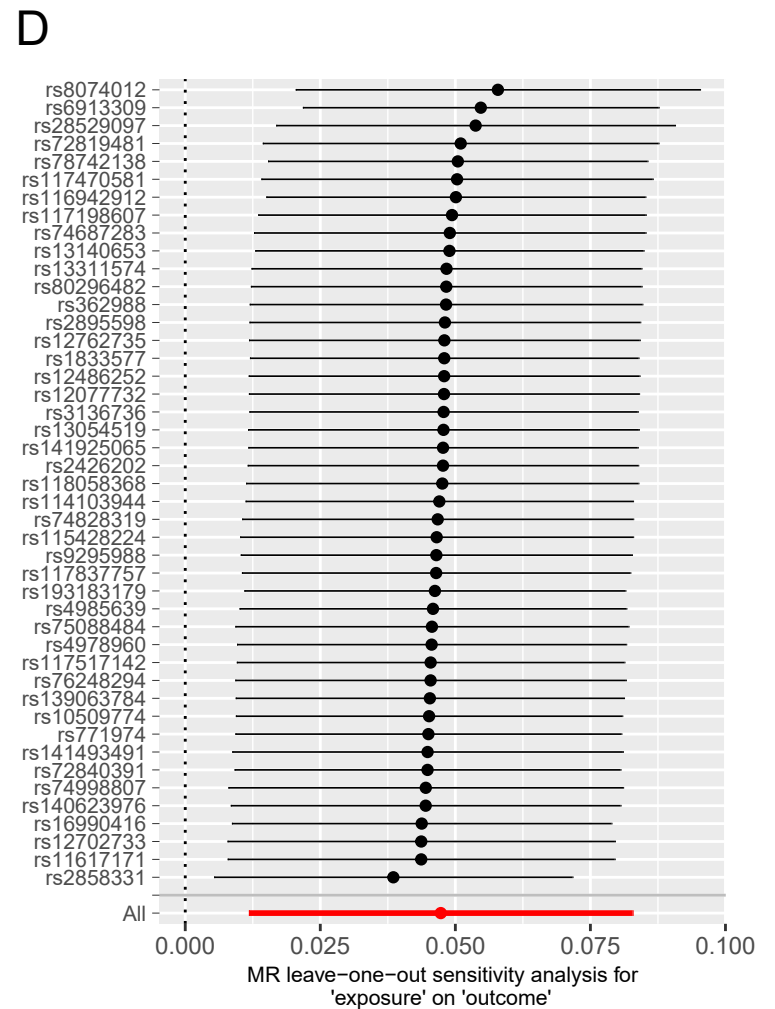

Supplement: Supplementary file 1 — Supporting Information 1 Supporting Figure 1: relationship examination between herpes keratitis and PD. (A) Funnel plot was used to illustrate the individual variation effects for the instrument variables shown against the inverse of their standard error. (B) Scatter plot depicted the causal relationship between herpes keratitis and PD by the line’s slope, which varies depending on the MR tests. (C) Forest plot was used to illustrate how herpes keratitis is associated with an increased risk of PD. (D) The causal association between herpes keratitis and PD is assessed by IVW approaches for each individual SNP. [file PADI-2025-4044371-s016.pdf]
