# Supplementary material for: Genome‐Wide Association Studies Data and Transcriptomics Data Link Herpes Simplex Virus 1 Infection and Parkinson’s Disease
Source: Parkinsons Dis. 2025 Dec 23;2025:4044371. doi: 10.1155/padi/4044371 (PMC12767429; doi:10.1155/padi/4044371)

exposure	outcome	method	nsnp	b	se	OR (95% CI)	P−value
Herpes Keratitis	PD	MR Egger	45	0.032590586	0.03059648	1.0331 (0.9730 − 1.0970)	0.2927
		Weighted median	45	0.004929171	0.02538992	1.0049 (0.9562 − 1.0562)	0.8461
		Simple mode	45	−0.003241350	0.05316420	0.9968 (0.8981 − 1.1062)	0.9517
		Weighted mode	45	−0.010321542	0.03195088	0.9897 (0.9297 − 1.0537)	0.7482

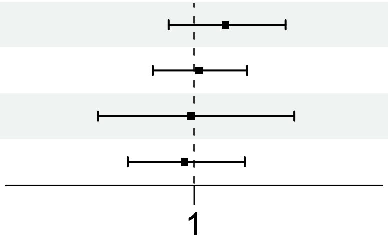

Supplement: Supplementary file 2 — Supporting Information 2 Supporting Figure 2: forest plot of MR results to test the causal relationship between herpes keratitis (exposure) and PD (outcome), using MR Egger, weighted median, simple mode, and weighted mode. [file PADI-2025-4044371-s001.pdf]
