# Supplementary material for: Genome‐Wide Association Studies Data and Transcriptomics Data Link Herpes Simplex Virus 1 Infection and Parkinson’s Disease
Source: Parkinsons Dis. 2025 Dec 23;2025:4044371. doi: 10.1155/padi/4044371 (PMC12767429; doi:10.1155/padi/4044371)

exposure	outcome	method	nsnp	b	se	OR (95% CI)	P−value
PD	Herpes Keratitis	MR Egger	112	0.015732583	0.06430337	1.0159 (0.8956 − 1.1523)	0.8072
		Weighted median	112	0.004704316	0.05347602	1.0047 (0.9047 − 1.1157)	0.9299
		Simple mode	112	0.029905047	0.12374907	1.0304 (0.8084 − 1.3132)	0.8095
		Weighted mode	112	−0.015231525	0.09092852	0.9849 (0.8241 − 1.1770)	0.8673

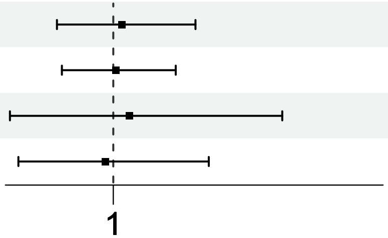

Supplement: Supplementary file 3 — Supporting Information 3 Supporting Figure 3: forest plot of MR results to test the causal relationship between PD (outcome) and herpes keratitis (exposure), using MR Egger, weighted median, simple mode, and weighted mode. [file PADI-2025-4044371-s002.pdf]
